# Docosahexaenoic Acid Enhances the Cytotoxic Effects of Enzalutamide and the PARP Inhibitor in Castration-Resistant Prostate Cancer Cell Lines

**DOI:** 10.3390/ijms27188037

**Published:** 2026-09-09

**Authors:** Alana Della Torre da Silva, Laurielle do Prado Ferreira, Daniele Lisboa Ribeiro, Guilherme Henrique Tamarindo, Rejane Maira Góes

**Affiliations:** 1 Department of Biological Sciences, Institute of Biosciences, Humanities and Exact Sciences, São Paulo State University (UNESP), São José do Rio Preto 15054-000, SP, Brazil; della.torre@unesp.br (A.D.T.d.S.); laurielle.prado@unesp.br (L.d.P.F.); 2 Department of Cell Biology, Histology and Embryology, Institute of Biomedical Sciences, Federal University of Uberlândia (UFU), Uberlândia 38405-318, MG, Brazil; daniele.ribeiro@ufu.br; 3 Brazilian Biosciences National Laboratory (LNBio), Brazilian Center for Research in Energy and Materials (CNPEM), Campinas 13083-100, SP, Brazil; guilherme.tamarindo@lnbio.cnpem.br

**Keywords:** castration-resistant prostate cancer (CRPC), docosahexaenoic acid (DHA), enzalutamide, PARP inhibitor, PC3 and 22Rv1 cells

## Abstract

Docosahexaenoic acid (DHA), an omega-3 fatty acid, has emerged as a promising adjuvant treatment for castration-resistant prostate cancer (CRPC), but its interactions with current therapies remain unclear. This study investigated the antitumor effects of DHA alone or in combination with enzalutamide (ENZ) or the PARP inhibitor AZD2461 (iP) in CRPC cell lines 22Rv1 and PC3. DHA enhanced the cytotoxic effect of iP in both cell lines and of ENZ in 22Rv1 cells. In PC3 cells, DHA-mediated potentiation of iP cytotoxicity was associated with downregulation of the AKT pathway, reduced expression of ERα and the lipid sensors PPARγ and LXRβ, and a shift in programmed cell death toward necroptosis. In 22Rv1 cells, the additive effects of DHA with ENZ or iP were independent of PI3K/pAKT and AR-FL/AR-V7 modulation but were associated with regulation of LXRβ, PPARγ, and TNFR1, along with activation of apoptosis. Wound-healing assays showed that DHA blocked the anti-migratory effects of ENZ and iP in 22Rv1 cells and did not alter iP effects in PC3 cells, indicating epithelial–mesenchymal transition heterogeneity. Overall, DHA potentiates the cytotoxic effects of iP in both models and of ENZ in 22Rv1 cells via differential modulation of cell death in a molecular context-dependent manner.

## 1. Introduction

Diet directly impacts health and can be used for the prevention and treatment of various human diseases, including cancer [1]. Prostate cancer (PCa) stands out as particularly sensitive to nutritional factors [2], especially regarding dietary lipids [1,2,3]. This disease is one of the major public health problems worldwide and affects millions of men annually [4] with a high incidence in developed Western countries, contrasting markedly with the low incidence found in developed Asian countries [3]. Beyond ethnic, hereditary, and environmental factors, this epidemiological disparity can be explained by dietary habits and the intake of omega-3 polyunsaturated fatty acids (ω-3 PUFAs) [3,5,6]. In addition, both the quantity and quality of dietary fats have changed profoundly worldwide in recent decades, characterized by reduced levels of ω-3 PUFAs and elevated levels of saturated fatty acids (SFAs), trans fatty acids (TFAs), and omega-6 PUFAs, which typify the Western dietary pattern [7,8].

Among ω-3 PUFAs, docosahexaenoic acid (DHA; C22:6 ω-3) has been extensively studied for its various beneficial health effects, including antitumoral properties [9,10,11,12,13,14,15,16,17]. Regarding PCa, whereas saturated fatty acids have been reported to drive disease progression either through diet or via increased expression of fatty acid synthase (FASN) in the castration-resistant stage [18], DHA has been shown to exert the opposite effect by reducing tumor growth in xenograft models or delaying progression in TRAMP mice [19,20,21]. In PCa, DHA can block the cell cycle, modulate proliferation and cell survival pathways, induce apoptosis, promote oxidative stress, cause mitochondrial dysfunction, alter lipid metabolism, modulate androgen signaling, and exert antiangiogenic and anti-inflammatory effects, among others [19,20,21,22,23,24,25,26,27]. Beyond classical apoptotic cell death mechanisms, recent studies have demonstrated that DHA possesses the capacity to modulate alternative regulated cell death pathways, including necroptosis and pyroptosis, which may be less susceptible to resistance mechanisms developed by tumor cells [28,29]. This ability of DHA to modulate multiple cell death pathways offers significant therapeutic advantages, particularly in contexts of tumor resistance where a single pathway may be compromised. Furthermore, this ω-3 fatty acid has been reported to preferentially target cancer cells while sparing normal cells in several models [17,30]. This differential sensitivity has been partly attributed to the lower antioxidant adaptability of tumor cells; additionally, metabolic differences between normal and malignant cells result in distinct processing and biological responses to DHA [17,30,31]. However, the effects of DHA on PCa still lack robust mechanistic studies for complete elucidation and, more importantly, clinical investigations, as current data remain conflicting [25].

The malignant transformation of the prostate is complex and follows a multistep process that may result in invasion of adjacent and distant organs, culminating in metastatic PCa [32]. Patients with PCa initially respond well to androgen deprivation therapy (ADT); however, treatment ultimately selects for ADT-resistant cancer cells, leading to the emergence of castration-resistant prostate cancer (CRPC), a more aggressive phenotype [33]. CRPC is defined as disease that progresses biochemically and/or clinically despite castrate levels of testosterone (<50 ng/dL). It is characterized by prostate-specific antigen (PSA) > 2 ng/mL with an increase of at least 25% above the nadir, confirmed by two consecutive measurements separated by ≥3 weeks, or by the appearance of ≥2 new lesions detectable on conventional imaging studies [34]. This disease represents a significant clinical challenge due to its incurable nature, with a median overall survival of only 1–2 years in metastatic cases, as well as the complexity of the resistance mechanisms involved [33,34]. Resistance develops through androgen receptor (AR)-dependent and AR-independent mechanisms. AR-dependent mechanisms include intratumoral androgen synthesis, *AR* gene amplification with increased expression at the mRNA and protein levels, expression of constitutively active splice variants (AR-Vs), such as AR-V7, and point mutations in the *AR* gene that confer activation by antiandrogens and non-androgenic steroids [33,34]. AR-independent mechanisms include neuroendocrine differentiation, growth factor signaling, involvement of inflammatory pathways that promote growth, and activation of alternative pathways, such as phosphoinositide 3-kinase/protein kinase B/mammalian target of rapamycin (PI3K/AKT/mTOR), which is frequently hyperactivated in CRPC [33,34].

The therapeutic landscape of PCa has undergone remarkable changes over the past decade. Several new classes of agents have emerged, including next-generation androgen receptor signaling inhibitors (ARSIs), such as enzalutamide (ENZ), and poly(ADP-ribose) polymerase (PARP) inhibitors (PARPi), such as olaparib. ENZ targets AR signaling at three principal levels: blocking androgen binding, inhibiting nuclear translocation, and impairing DNA binding [35,36]. Despite its high affinity for AR, ENZ efficacy is limited by the development of resistance mediated by multiple interconnected mechanisms, notably the emergence of AR-V7 [35,36]. Other mechanisms include *AR* gene amplification and mutations, compensatory glucocorticoid receptor (GR) activation, metabolic and epigenetic alterations, and autophagy-mediated resistance, which frequently coexist and justify the necessity for combined and personalized therapeutic strategies [37,38]. PARPi represent a therapeutic standard in CRPC for patients who have progressed following prior therapy and who harbor mutations in homologous recombination repair (HRR) genes, such as *BRCA1*, *BRCA2*, and *ATM* [3]. They act through two complementary mechanisms: catalytic inhibition and PARP trapping [39]. In catalytic inhibition, they compete with nicotinamide adenine dinucleotide (NAD^+^) for the active catalytic site, thus preventing the synthesis of poly(ADP-ribose) polymers and PARylation, thereby leading to accumulation of single-strand DNA breaks (SSBs), which, if unrepaired, convert into double-strand breaks (DSBs) [39]. They are also capable of trapping PARP at damage sites, generating a physical barrier that prevents the access of other repair proteins [39]. This results in lethal DNA damage in HRR-deficient tumors while sparing normal cells [40]. Despite their efficacy, the emergence of resistance prompted the development of next-generation compounds such as AZD2461, which was developed as a successor to olaparib, the first PARPi approved for cancer therapy [41]. AZD2461 exhibits reduced sensitivity to drug resistance mechanisms, as it is a poor substrate for the P-glycoprotein (P-gp) efflux pump, in addition to displaying greater selectivity for proliferating cells with DSB repair dysfunction, enhanced tolerability in combination with chemotherapy, and the capacity to induce cell death even in olaparib-resistant cell lines [42].

Despite recent therapeutic advances, resistance to treatment remains the principal obstacle in CRPC management, highlighting the need for innovative strategies that can overcome these resistance mechanisms and improve clinical outcomes [43]. In this context, DHA emerges as a promising therapeutic alternative, not only for its classical antitumoral mechanisms, but particularly for its potential to modulate resistance pathways developed by tumor cells against conventional therapies [44,45]. Additionally, DHA possesses the capacity to sensitize tumor cells to anticancer agents through modulation of multiple molecular pathways, including alterations in cell membrane fluidity, modulation of androgen signaling, and reduction in mitochondrial spare respiratory capacity and the ability to respond to cellular stress [26], potentially offering synergistic benefit when combined with targeted therapies [19,25,44,46]. Here, we hypothesized that DHA, in combination with drugs used in CRPC treatment, could enhance cytotoxic effects in castration-resistant cells through mechanisms independent of those targeted by these drugs. Thus, this study investigated the antitumoral effects of DHA alone and in combination with enzalutamide and the PARPi AZD2461 in CRPC cell lines, evaluating mechanisms of cell death induction, including apoptosis, necroptosis, and pyroptosis, as well as characterizing potential enhanced effects of these combinations, thereby contributing to the development of innovative and more efficacious therapeutic strategies for the treatment of CRPC.

## 2. Results

### 2.1. DHA Enhances the iP-Induced Mitochondrial Metabolic Rate Impairment in PC3 Cells but Not in 22Rv1 Cells

First, we investigated whether DHA could potentiate the effects of enzalutamide (ENZ) and the PARP inhibitor AZD2461 (iP), based on mitochondrial metabolic rate (MMR) alterations in AR-positive (22Rv1) and AR-negative (PC3) prostate cancer cells. This approach allowed us to identify treatment conditions that not only modulate metabolic activity but also capture cell line-specific vulnerabilities that could underlie subsequent cytotoxic responses.

**Figure 1:**
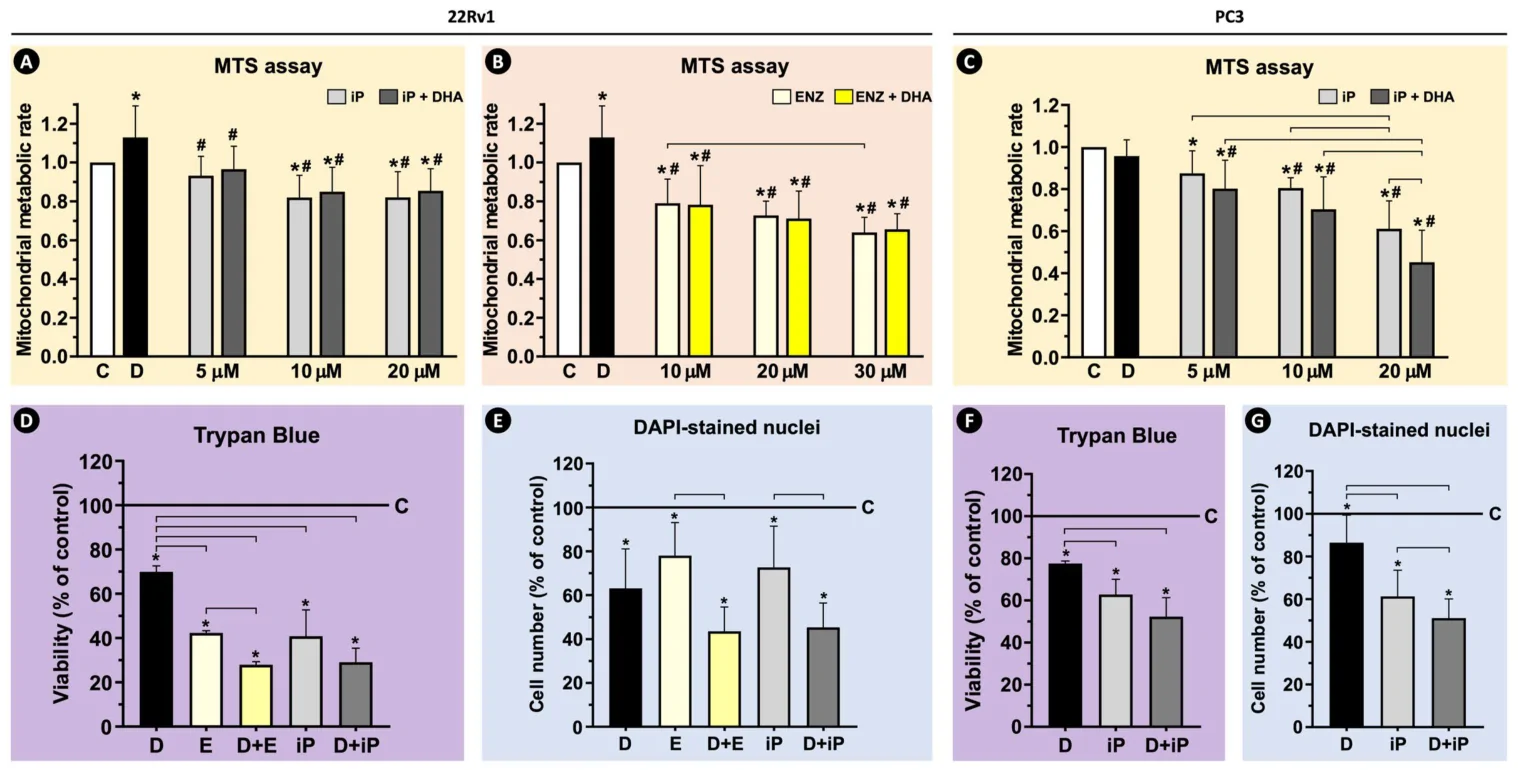
Cytotoxic effects of DHA alone or in combination with enzalutamide (ENZ) and the PARP inhibitor AZD2461 (iP) in 22Rv1 and PC3 cells. Cells were treated for 48 h with the indicated compounds. (**A**–**C**) MTS assay in 22Rv1 cells (**A**,**B**) and PC3 cells (**C**), indicating mitochondrial metabolic rate following 48 h of treatment with the indicated concentrations of iP, ENZ, and DHA (100 μM). (**D**,**F**) Trypan blue exclusion assay in 22Rv1 (**D**) and PC3 cells (**F**), expressed as the percentage of viable cells relative to the control after treatment with DHA (100 μM), ENZ (30 μM), and iP (20 μM). (**E**,**G**) Quantification of cell number after nuclear staining with DAPI, expressed as the percentage of cells relative to control in 22Rv1 (**E**) and PC3 cells (**G**). Data are expressed as mean ± standard deviation from four independent experiments (*n* = 4). Asterisks indicate significant differences compared with the control group, and hash symbols indicate significant differences compared with the DHA group (*p* < 0.05). Square brackets indicate statistically significant differences between experimental groups (*p* < 0.05). Parametric data: MTS and trypan blue assay. Non-parametric data: DAPI-stained nuclei. C = Control; D = DHA (100 μM); E = Enzalutamide (30 μM); iP = PARP inhibitor AZD2461 (20 μM); D+E = DHA + Enzalutamide; D+iP = DHA + PARP inhibitor AZD2461.

For both cell lines, the most pronounced alterations in MMR were observed after 48 h of incubation. In 22Rv1 cells, DHA (100 μM) slightly increased MMR, whereas iP reduced MMR at concentrations of 10 and 20 μM. ENZ induced a dose-dependent reduction, with the greatest effect observed at 30 μM (37%). However, combining DHA with either ENZ or iP did not further decrease MMR compared with the corresponding monotherapies (Figure 1A,B). In PC3 cells, DHA did not alter MMR, whereas iP reduced MMR in a dose-dependent manner. Notably, co-treatment with DHA potentiated the effect of iP, with the greatest reduction observed for the DHA + 20 μM iP combination (55%), indicating that the fatty acid potentiated the effect of the drug (Figure 1C).

Based on these results, treatment conditions of 20 μM iP and 30 μM ENZ for 48 h were selected for all subsequent experiments.

### 2.2. DHA Enhances the Cytotoxicity of ENZ and iP

Having established that DHA modulates MMR in a cell line-dependent manner and considering our previous report showing that this lipid affects mitochondrial cell metabolism [24], we next investigated whether these alterations were accompanied by meaningful effects on cell viability and cell number. By combining Trypan Blue exclusion and automated nuclear counting, we aimed to discriminate between changes in metabolic activity and bona fide reductions in proliferative capacity and survival under mono- and combination therapies.

In both cell lines, all treatments significantly reduced cell viability compared with the control (Figure 1D,F). In 22Rv1 cells, co-treatment with DHA further enhanced ENZ-induced cytotoxicity, resulting in the greatest reduction in cell viability (72%). In contrast, although DHA+iP also markedly reduced viability (70%), it did not differ significantly from iP alone. Similarly, in PC3 cells, DHA and iP decreased cell viability, but their combination did not further enhance this effect (Figure 1F). Automated cell counting confirmed these findings (Figure 1E,G). All treatments reduced cell number in both cell lines compared with the control. In 22Rv1 cells, DHA potentiated the effects of both ENZ and iP, resulting in significantly fewer cells than either drug alone. Likewise, in PC3 cells, the DHA+iP combination produced the greatest reduction in cell number (49%) suggesting an additive cytotoxic effect (Figure 1G).

### 2.3. DHA Potentiates the Inhibition of the AKT Pathway Induced by iP in PC3 Cells

**Figure 2:**
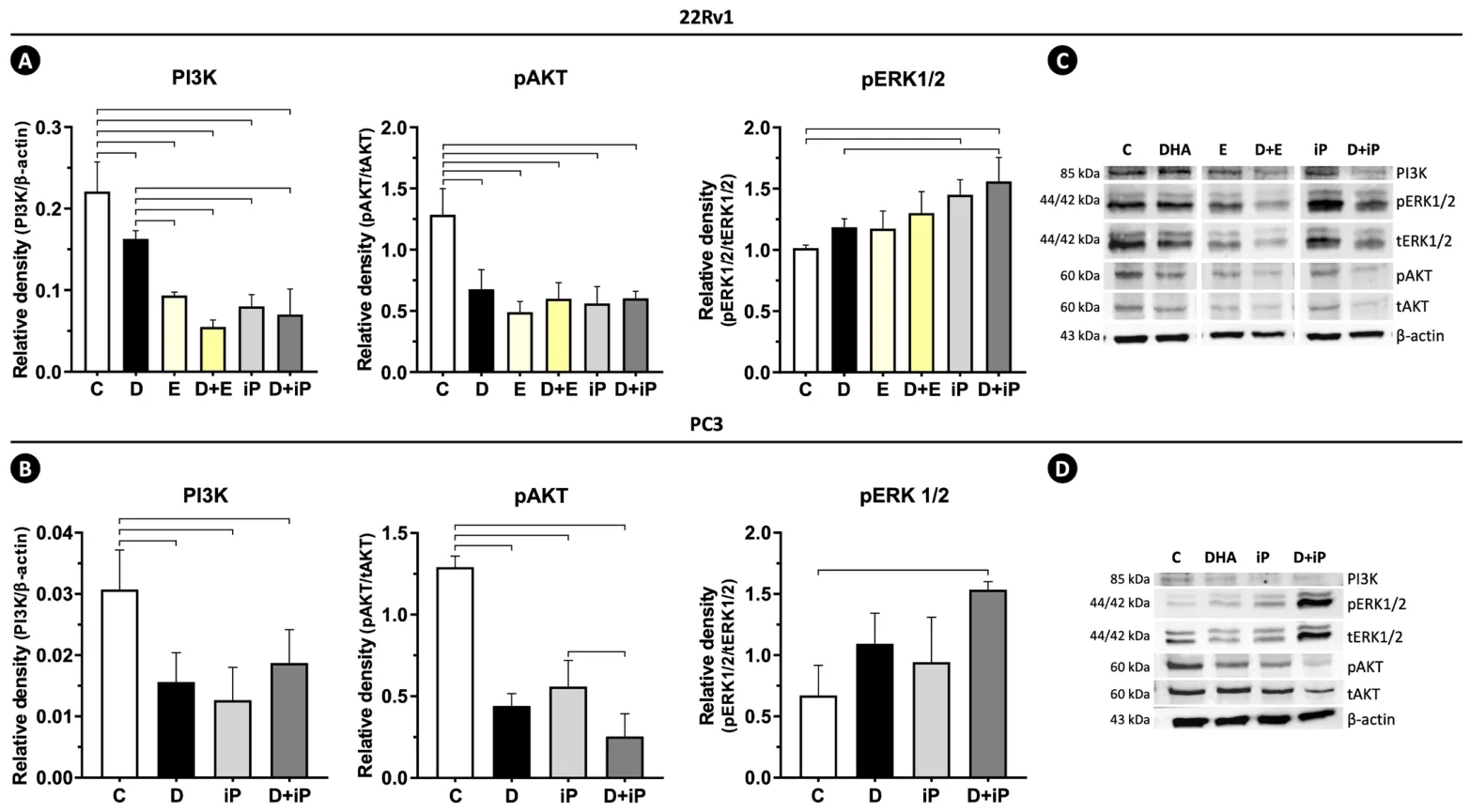
Response of proliferation and cell survival pathway markers following treatment with DHA alone or combined with enzalutamide or with the PARP inhibitor AZD2461 in 22Rv1 and PC3 cells. Cells were treated for 48 h with the indicated compounds, and protein levels from cell extracts were analyzed by Western blotting. (**A**,**B**) Quantification of protein levels of PI3K, pAKT, pERK1/2 in 22Rv1 ((**A**), upper panels) and PC3 cells ((**B**), lower panels). (**C**,**D**) Representative immunoblots of the indicated proteins in 22Rv1 (**C**) and PC3 cells (**D**). Values represent relative density. Protein levels were normalized to β-actin or total protein, as appropriate. A representative β-actin blot is shown for illustrative purposes. Data are expressed as mean ± standard deviation from four independent experiments (*n* = 4). Square brackets indicate statistically significant differences between experimental groups (*p* < 0.05). Parametric data: pAKT; PI3K (PC3); pERK1/2 (PC3). Non-parametric data: PI3K (22Rv1); pERK1/2 (22Rv1). C = Control; D = DHA (100 μM); E = Enzalutamide (30 μM); iP = PARP inhibitor AZD2461 (20 μM); D+E = DHA + Enzalutamide; D+iP = DHA + PARP inhibitor AZD2461.

Considering the cell viability effects, we investigated whether the primary cell survival and growth pathways (PI3K/AKT and ERK) were affected. These pathways are central to the regulation of cell growth, proliferation, and survival, and are frequently dysregulated in cancer [47]. Western blotting assays demonstrated that all treatments reduced the expression of proliferation and cell survival pathway markers, PI3K and phosphorylated AKT (pAKT), in both cell lines compared with the control (Figure 2A–D). In 22Rv1 cells, these markers did not differ among the groups treated in monotherapy or in combination with DHA (Figure 2A,C); however, in PC3 cells pAKT was further reduced in the DHA+iP group compared with iP (Figure 2B,D). Furthermore, incubation with iP alone or in combination with DHA increased phosphorylated ERK1/2 (pERK1/2) expression in 22Rv1 cells, whereas in PC3 cells this increase was observed only with DHA+iP co-incubation (Figure 2A–D).

### 2.4. DHA Reduces AR-FL but Does Not Affect AR-V7 in 22Rv1 Cells

**Figure 3:**
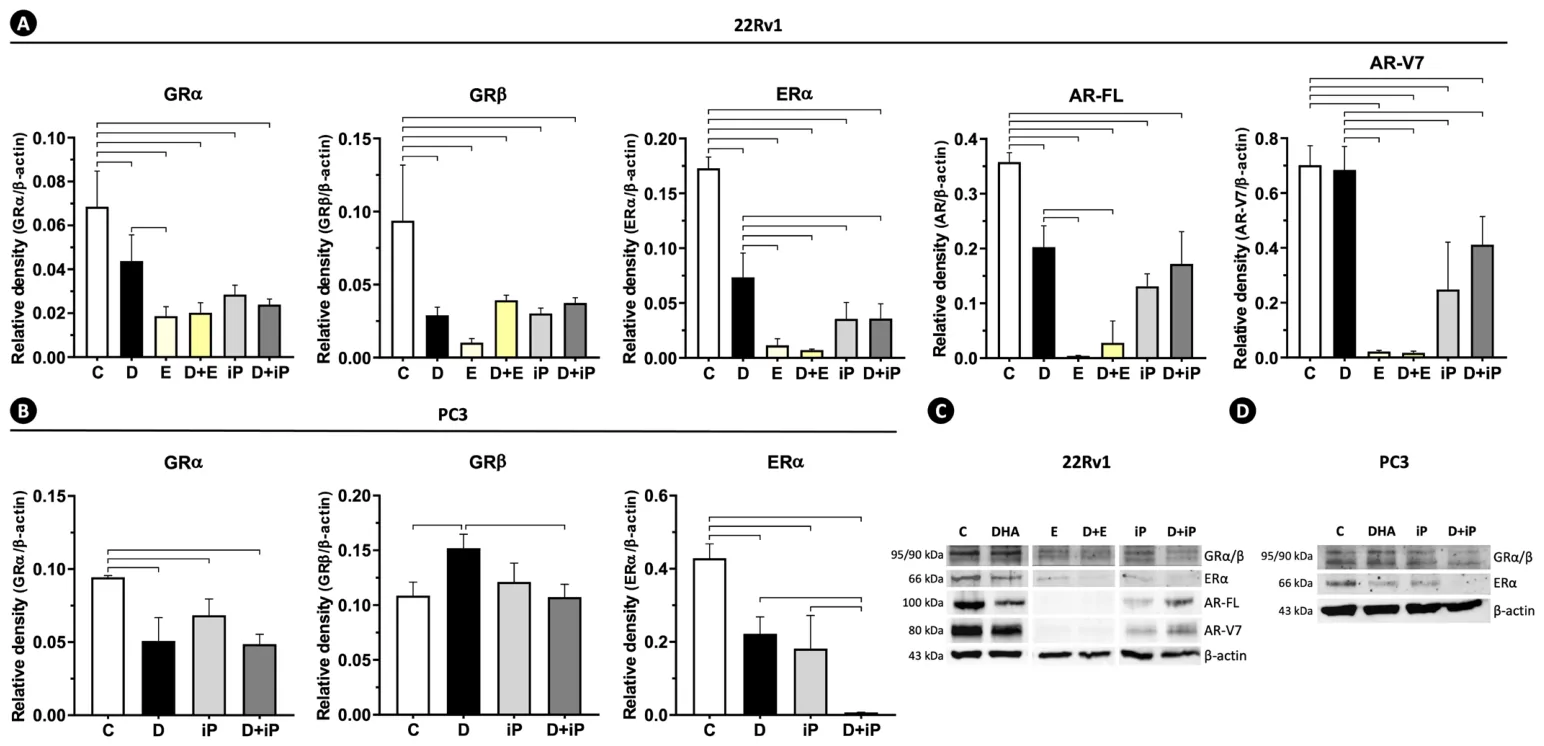
Response of nuclear receptors related to escape mechanisms, therapeutic resistance, and proliferation in CRPC cells following treatment with DHA alone or in combination with enzalutamide or the PARP inhibitor AZD2461. Cells were treated for 48 h with the indicated compounds, and protein levels from cell extracts were analyzed by Western blotting. (**A**,**B**) Quantification of protein levels of GRα, GRβ, and ERα in 22Rv1 ((**A**), upper panels) and PC3 cells ((**B**), lower panels). Quantification of protein levels of AR full-length and AR variant 7 (AR-V7, only in 22Rv1 cells). (**C**,**D**) Representative immunoblots of the indicated proteins in 22Rv1 (**C**) and PC3 cells (**D**). Values represent relative density. Protein levels were normalized to β-actin. A representative β-actin blot is shown for illustrative purposes. Data are expressed as mean ± standard deviation from four independent experiments (*n* = 4). Square brackets indicate statistically significant differences between experimental groups (*p* < 0.05). Parametric data: GR and ERα. Non-parametric data: AR. C = Control; D = DHA (100 μM); E = Enzalutamide (30 μM); iP = PARP inhibitor AZD2461 (20 μM); D+E = DHA + Enzalutamide; D+iP = DHA + PARP inhibitor AZD2461.

Given that 22Rv1 cells express both AR-FL and constitutively active variants, such as AR-V7, we next evaluated whether DHA and its combinations with ENZ and iP could interfere with this signaling axis. Discriminating between the effects on AR-FL and AR-V7 is crucial, as AR-V7 is a major driver of resistance to next-generation antiandrogens. All treatments reduced full-length androgen receptor (AR-FL) expression in 22Rv1 cells compared with the control, with reductions of 99% and 92% in the ENZ and DHA+ENZ groups, respectively, without statistical differences between them. DHA did not alter AR-V7 variant expression; however, all other treatments reduced its expression, with the most pronounced reductions observed in ENZ-treated groups (Figure 3A,C). AR was not evaluated in PC3 cells as this cell line does not express the receptor.

### 2.5. ENZ and iP Reduce GRα/ERα Expression in Both Cell Lines, and DHA Enhances ERα Downregulation in PC3 Cells

Escape from AR dependence in CRPC frequently involves compensatory activation of alternative nuclear receptors, including GR and ERα. In this context, we next interrogated how DHA and its combinations with ENZ and iP affect these receptors related to escape mechanisms, therapeutic resistance, and proliferation in CRPC. Mapping GRα/β and ERα expression in both 22Rv1 and PC3 cells allowed us to evaluate whether DHA acts solely through classical AR-related mechanisms or also reshapes wider steroid receptor networks.

All treatments reduced the expression of GRα and GRβ isoforms in 22Rv1 cells (Figure 3A,C). In PC3 cells, all treatments reduced GRα expression; however, GRβ increased by 40% with DHA (Figure 3B,D). Estrogen receptor α (ERα) showed a pronounced reduction with all treatments in both 22Rv1 and PC3 cells (Figure 3). In PC3 cells, the DHA+iP combination resulted in an additional 96% reduction in ERα compared with iP, indicating an enhanced effect of the combination (Figure 3B,D).

### 2.6. DHA Drastically Inhibits Colony Growth

**Figure 4:**
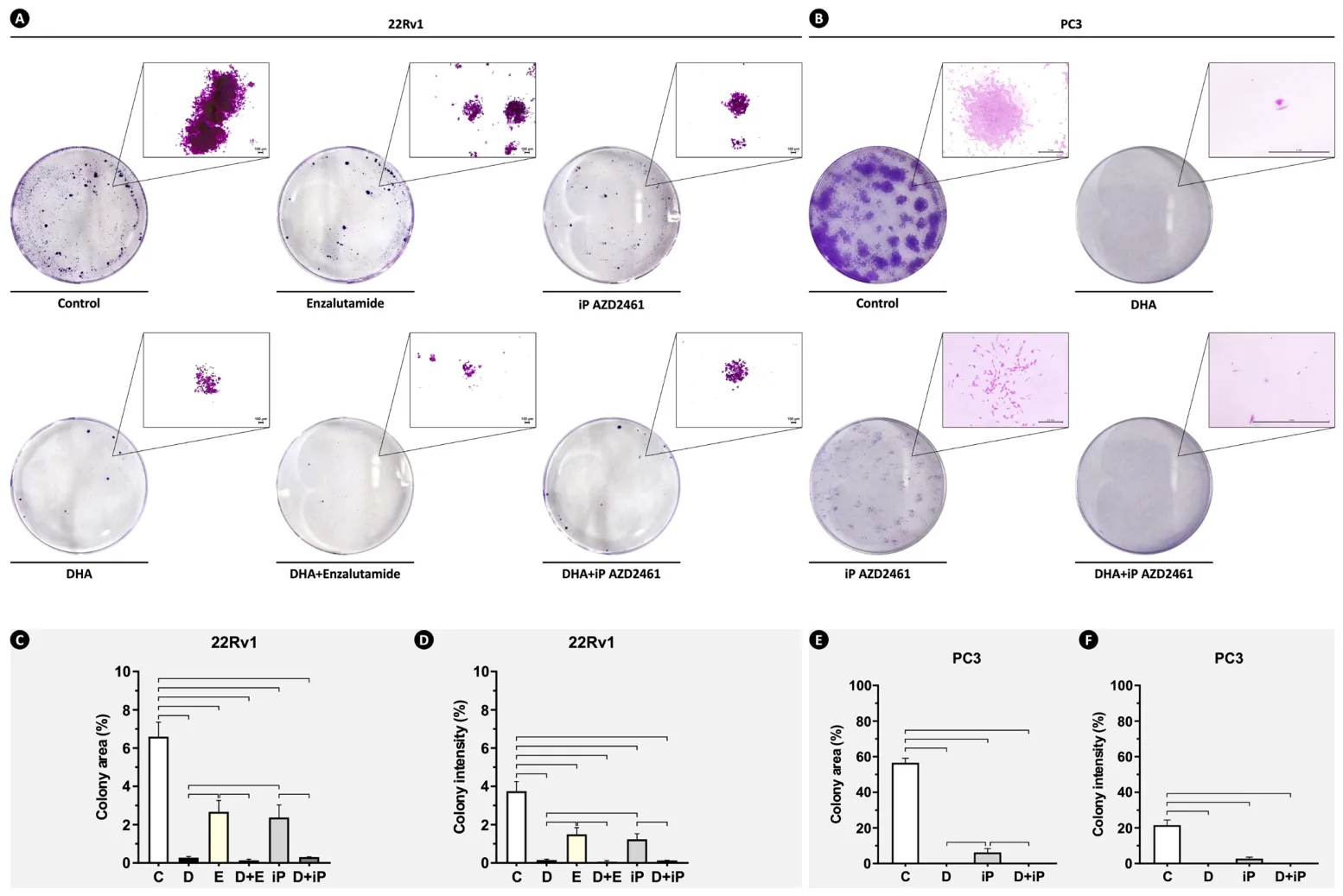
Anti-clonogenic effects of DHA alone or combined with enzalutamide (ENZ) and the PARP inhibitor AZD2461 (iP) on 22Rv1 and PC3 cells. (**A**,**B**) Representative images of clonogenic assay plates showing cell survival and proliferative capacity after 14 days, following 48 h of treatment (DHA 100 μM, ENZ 30 μM, and iP 20 μM—48 h) in 22Rv1 (**A**) and PC3 (**B**) cells. Staining: Crystal Violet. (**C**,**E**) Quantification of percentage colony area and (**D**,**F**) colony intensity from clonogenic assays in 22Rv1 (**C**,**D**) and PC3 (**E**,**F**) cells. Data are represented as mean ± standard deviation. Square brackets indicate statistically significant differences (*p* < 0.05). The 22Rv1 cell line data followed a parametric distribution, while the PC3 data are non-parametric. C = Control, D = DHA (100 μM), E = Enzalutamide (30 μM), iP = PARP inhibitor AZD2461 (20 μM), D+E = DHA + Enzalutamide, D+iP = DHA+PARP inhibitor AZD2461.

To move beyond short-term viability and quantify the long-term clonogenic potential of CRPC cells, we next assessed the impact of DHA and drug combinations on colony growth. Clonogenic assays are particularly informative in this setting because they capture the ability of surviving cells to proliferate and repopulate after treatment, a key feature of therapy resistance and disease relapse. 22Rv1 cells occupied a smaller area of the well compared to PC3 cells; however, colony intensity was proportionally greater, indicating the capacity of these cells to grow densely (Figure 4). DHA exerted a stronger inhibitory effect on colony growth in 22Rv1 cells, reducing the occupied area by 96%, whereas ENZ and iP reduced it by 59% and 64%, respectively, compared to control. The DHA+ENZ and DHA+iP combinations dramatically reduced the area occupied by colonies (98% and 95%, respectively), similar to the effect of DHA (Figure 4A,C,D). Colony intensity also decreased, indicating an impact on the proliferative capacity of cells within colonies. DHA treatments completely inhibited PC3 cell colony formation, with only isolated cells remaining. In contrast, iP treatment resulted in an 89% reduction in colony area and an 87% reduction in colony intensity compared to control (Figure 4B,E,F).

### 2.7. DHA Impairs EMT and Migration in 22Rv1 Cells Under ENZ and iP, with Differential Effects in PC3 Cells

Given the central role of EMT and migratory capacity in metastatic spread and therapeutic resistance in CRPC, we next evaluated whether DHA modulated some indicators of these processes under ENZ and iP treatment. By integrating wound healing assays with EMT marker profiling, we aimed to determine whether the observed changes in viability, signaling, and receptor status translate into altered migratory behavior.

**Figure 5:**
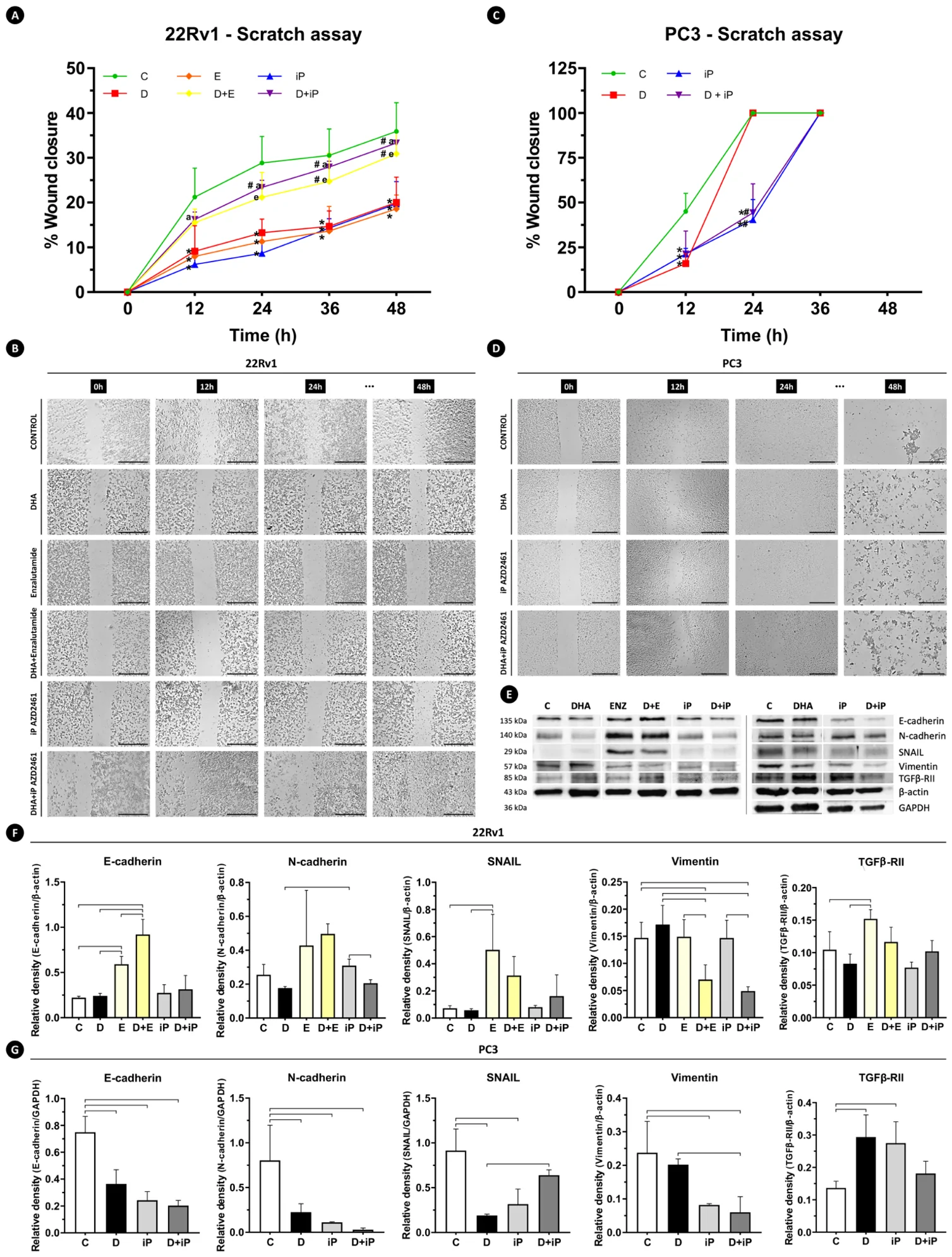
Effects of DHA, enzalutamide, and PARP inhibitor AZD2461 on cell migration and epithelial–mesenchymal transition in CRPC cell lines. 22Rv1 and PC3 cells were treated for 48 h with DHA (100 µM; D), enzalutamide (30 µM; E), PARP inhibitor AZD2461 (20 µM; iP), DHA+enzalutamide (D+E), or DHA+AZD2461 (D+iP) (C = control). (**A**,**C**) Quantification of wound healing assay (scratch assay) showing wound closure over time (0, 12, 24, 36, and 48 h) following the indicated treatments in 22Rv1 (**A**) and PC3 (**C**) cells. The 48 h time point in PC3 cells was not used for migration interpretation due to evident loss of monolayer integrity. (**B**,**D**) Representative wide-field images of the wound area at the indicated time points in 22Rv1 (**B**) and PC3 (**D**) cells. Scale bars = 1000 µm. (**E**) Western blotting analysis (representative immunoblots) of epithelial–mesenchymal transition (EMT) marker expression in 22Rv1 cells (left) and PC3 cells (right). (**F**,**G**) Densitometric quantification of E-cadherin, N-cadherin, SNAIL, vimentin, and TGFβ-RII in 22Rv1 (**F**) and PC3 (**G**) cells. A representative β-actin blot is shown for illustrative purposes. Statistics: data are expressed as mean ± standard deviation. * < 0.05 vs. control; # < 0.05 vs. DHA; e < 0.05 vs. enzalutamide; a < 0.05 vs. PARP inhibitor AZD2461. Square brackets indicate statistically significant differences between experimental groups (*p* < 0.05). Parametric data: N-cadherin, Vimentin, E-cadherin (22Rv1), SNAIL (22Rv1), scratch assay (22Rv1), TGFβ-RII (PC3). Non-parametric data: E-cadherin (PC3), SNAIL (PC3), scratch assay (PC3), TGFβ-RII (22Rv1). Cell images are displayed in grayscale with 100% clarity to evidence cell localization.

The wound healing assay in 22Rv1 cells indicated that single treatments inhibited cell migration similarly, resulting, at 48 h, in wound closure of 19% for ENZ and 20% for iP and DHA, whereas control cells reached 36% closure. Wound closure after DHA+ENZ and DHA+iP combinations did not differ from control at the analyzed time points (Figure 5A,B). In PC3 cells, wound closure kinetics were substantially different from those observed in 22Rv1 cells. The control group showed a rapid reduction in the initial wound area, reaching approximately 45% at 12 h. At 24 h, the original continuous wound region was no longer clearly distinguishable; however, small discontinuous cell-free areas and reduced cellular density remained within the region initially occupied by the scratch. A similar pattern was observed after DHA treatment. DHA monotherapy initially delayed wound closure, reaching approximately 16% closure at 12 h. iP monotherapy and DHA+iP delayed wound closure in PC3 cells, with approximately 21% closure at 12 h and 40% and 44% closure at 24 h, respectively, followed by complete closure by 36 h (Figure 5C,D). At 48 h, loss of monolayer integrity, including cell death and cell detachment, was observed in control cells and, to a lesser extent, in DHA-treated cells. Therefore, the 48 h time point was not considered suitable for interpreting PC3 cell migration under these conditions.

Western blotting analysis of epithelial–mesenchymal transition (EMT) markers of 22Rv1 cells (Figure 5E,F) demonstrated a 2.7-fold increase in E-cadherin expression in the ENZ group and a 4.1-fold increase in the DHA+ENZ group compared to control, with DHA potentiating the ENZ-induced increase. N-cadherin showed no variation in any group compared to control; however, it was reduced 1.5-fold in the DHA+iP group compared to iP. SNAIL increased only in the ENZ group compared to control. Vimentin expression was reduced in the DHA+ENZ (~52%) and DHA+iP (67%) groups compared to control. It also decreased compared to DHA and the monotherapy drugs. Transforming growth factor β receptor II (TGFβ-RII) increased only in the ENZ group (~45%) (Figure 5E,F). In PC3 cells (Figure 5E,G), E-cadherin and N-cadherin expression decreased in all treatments compared to control. SNAIL decreased in DHA and iP, but not in DHA+iP. Vimentin expression decreased in groups treated with iP alone (~65%) or in combination (DHA+iP, ~75%) compared to control. TGFβ-RII increased in DHA (115%) and iP (102%) (Figure 5E,G).

### 2.8. DHA Modulates LC3B Accumulation in 22Rv1 and PC3 Cells Through PPARγ and mTOR Signaling

**Figure 6:**
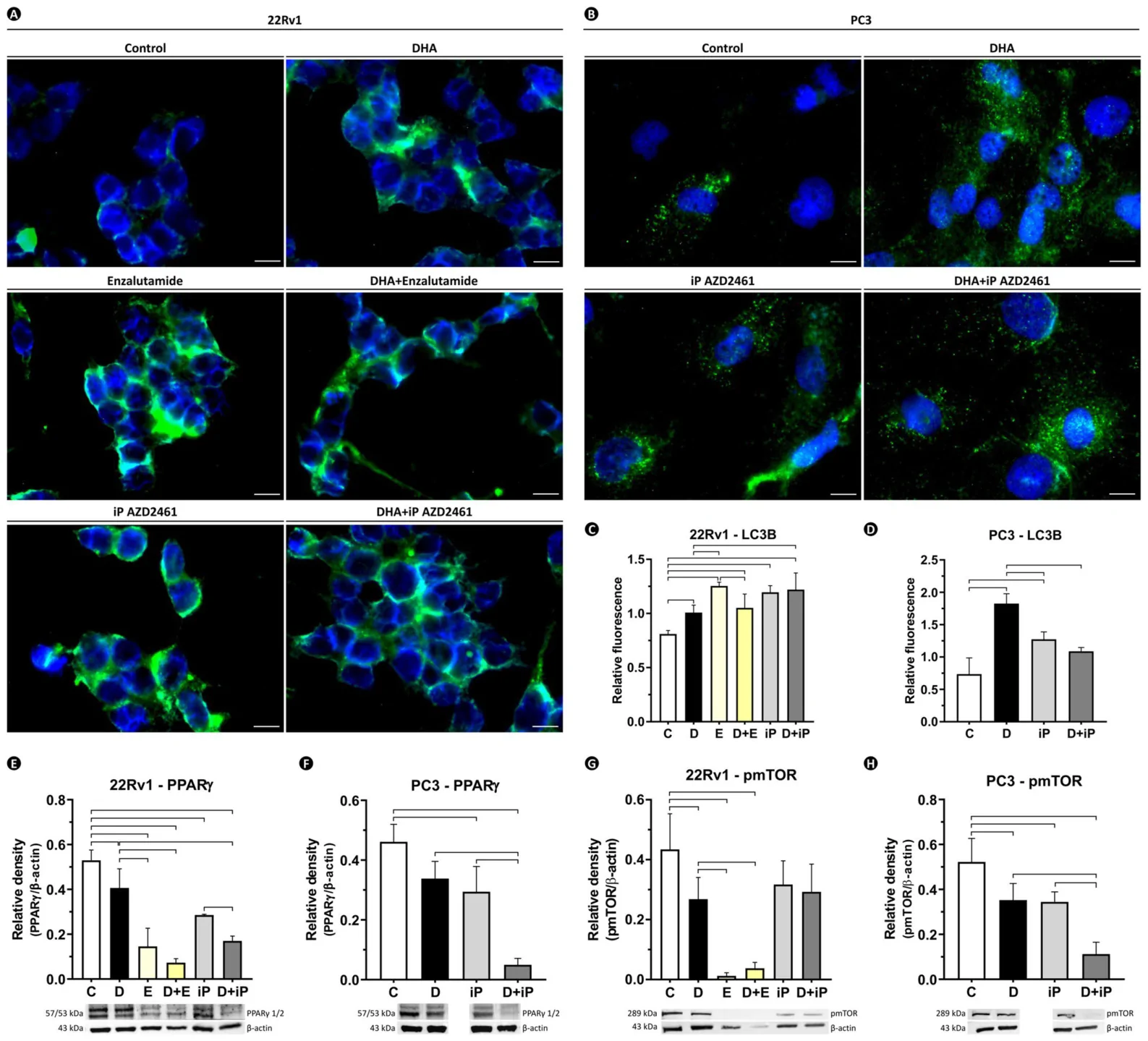
Effects of treatment with DHA, enzalutamide, and PARP inhibitor AZD2461, alone or in combination, on LC3B accumulation and metabolic signaling in CRPC cells. (**A**,**B**) Representative immunofluorescence images showing cytoplasmic LC3B (autophagosomes/autophagy marker, green) and nuclei (DAPI, blue) in 22Rv1 (**A**) and PC3 (**B**) cells. (**C**,**D**) Quantification of relative LC3B fluorescence intensity in 22Rv1 (**C**) and PC3 (**D**) cells. (**E**,**F**) Western blotting analysis of PPARγ protein levels in 22Rv1 (**E**) and PC3 (**F**) cells. PPARγ mainly regulates transcriptional programs associated with metabolic homeostasis and lipid handling. (**G**,**H**) Western blotting analysis of phospho-mTOR (pmTOR) levels in 22Rv1 (**G**) and PC3 (**H**) cells, indicative of mTOR pathway activity and autophagy regulation. Data are expressed as mean ± standard deviation. Square brackets indicate statistically significant differences (*p* < 0.05). Parametric data: pmTOR, PPARγ (PC3), LC3B (PC3). Non-parametric data: PPARγ (22Rv1), LC3B (22Rv1). Scale bars = 20 μm. C = Control; D = DHA (100 μM); E = Enzalutamide (30 μM); iP = PARP inhibitor AZD2461 (20 μM); D+E = DHA + Enzalutamide; D+iP = DHA+PARP inhibitor AZD2461.

Immunofluorescence analysis revealed that LC3B fluorescence labeling increased in 22Rv1 cells following all treatments compared with the control. LC3B fluorescence labeling was lower in the DHA+ENZ than in the ENZ group, indicating that DHA attenuated the ENZ-induced increase in LC3B levels (Figure 6A,C). In PC3 cells, DHA markedly increased LC3B fluorescence labeling by 148%, whereas iP induced a more moderate increase (73%). Notably, the DHA+iP combination restored LC3B fluorescence intensity to levels comparable to those of the control (Figure 6B,D).

To explore signaling pathways potentially associated with the observed changes in LC3B accumulation, we next evaluated the protein levels of PPARγ and phosphorylated mTOR (pmTOR). PPARγ was selected because of its reported role in regulating the expression of autophagy-related genes, including *MAP1LC3B* (LC3B), whereas mTOR is a major negative regulator of autophagosome formation. In 22Rv1 cells, PPARγ content decreased after DHA, ENZ, and iP, and a greater reduction was found in DHA+iP compared with iP alone (~41%) (Figure 6E). In PC3 cells, DHA did not alter PPARγ content, while iP reduced its expression by ~36%, and its combination with DHA reduced it by ~89% (Figure 6F). Regarding pmTOR protein levels, in 22Rv1 cells, DHA reduced pmTOR expression by 38%, whereas ENZ-containing treatments reduced pmTOR levels by more than 90% relative to the control. Treatments with iP did not alter pmTOR in this cell line (Figure 6G). In PC3 cells, DHA and iP caused similar reductions in pmTOR levels (~34%) compared with control, whereas their combination resulted in a 78% decrease relative to control, with pmTOR levels approximately 3.1-fold lower than those observed with iP alone (Figure 6H).

### 2.9. Treatments Induce Apoptosis Through Cell Line-Dependent Mechanisms

To clarify whether the reductions in viability, clonogenicity, and signaling pathways were accompanied by activation of classical programmed cell death, we next analyzed apoptotic markers, including activated caspase-3, cleaved PARP (cPARP), and BCL2.

**Figure 7:**
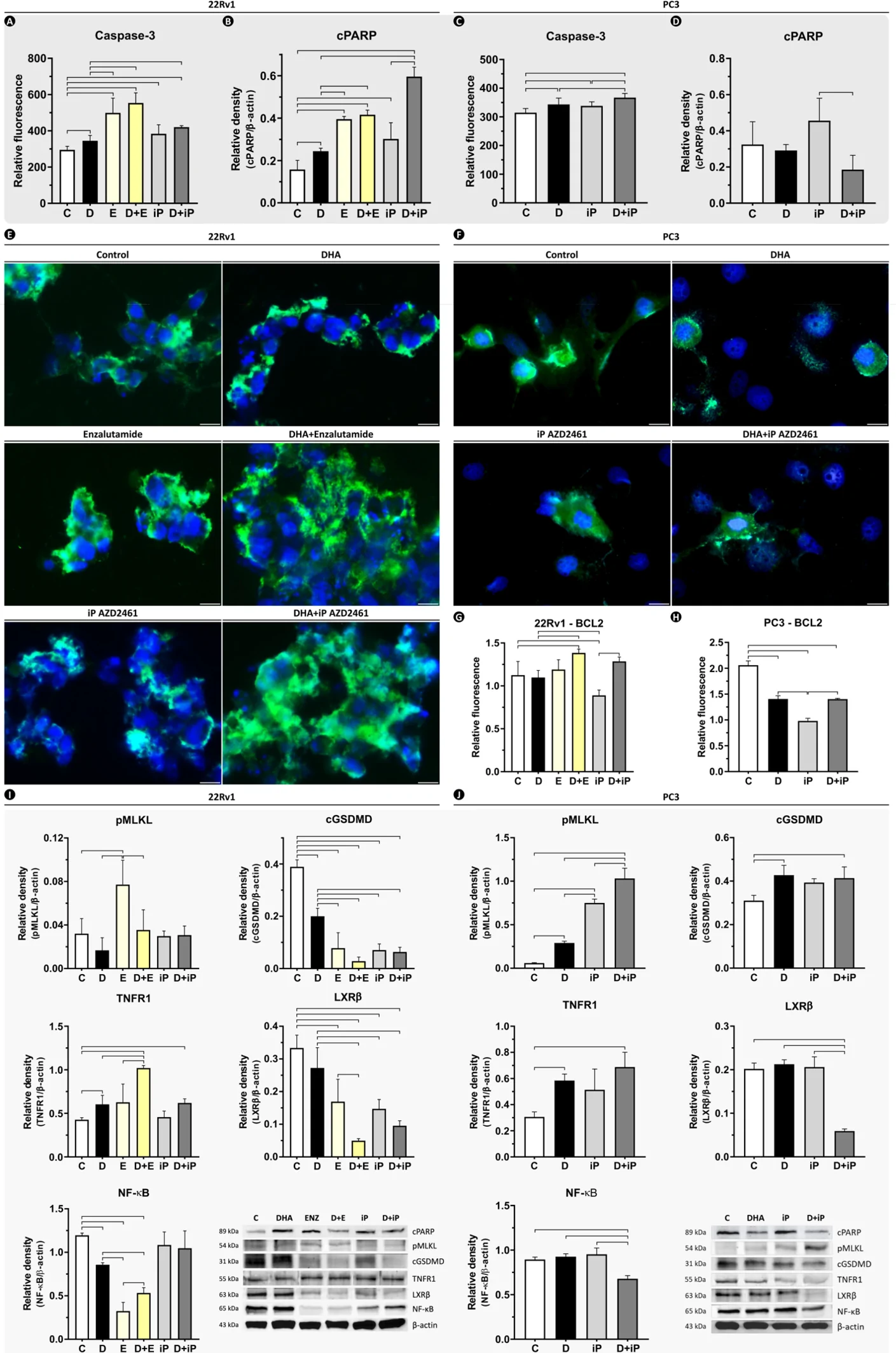
Response of different regulated cell death pathways following treatment with DHA alone or combined with enzalutamide or PARP inhibitor AZD2461 in representative CRPC cell lines. 22Rv1 and PC3 cells were treated for 48 h with DHA (100 µM; D), enzalutamide (30 µM; E), PARP inhibitor AZD2461 (20 µM; iP), and their combinations (D+E and D+iP). (**A**,**C**) Caspase-3 proteolytic activity in 22Rv1 cells (**A**) and PC3 cells (**C**). Caspase-3 activity was evaluated using a fluorimetric assay with Z-DEVD-AMC substrate, normalized to protein concentration. (**B**,**D**) Western blotting showing levels of cleaved PARP as a marker of apoptosis in 22Rv1 cells (**B**) and PC3 cells (**D**). (**E**,**F**) Immunofluorescence for localization and expression of the antiapoptotic protein BCL2 in 22Rv1 cells (**E**) and PC3 cells (**F**). Images show predominantly cytoplasmic localization of BCL2 (green), with nuclei counterstained with DAPI (blue). (**G**,**H**) Quantification of relative BCL2 fluorescence normalized to mean nuclear fluorescence intensity in 22Rv1 cells (**G**) and PC3 cells (**H**). (**I**,**J**) Western blotting analyzing markers of alternative regulated cell death pathways in 22Rv1 cells (**I**) and PC3 cells (**J**), followed by representative immunoblots. β-actin was used as endogenous control. A representative β-actin blot is shown for illustrative purposes. (**I**,**J**) (**Upper panel**) pMLKL indicates necroptosis execution and cleaved GSDMD indicates pyroptosis. (**Middle panel**) TNFR1, LXRβ, receptors associated with regulation of these cell death modes. (**Lower panel**) NF-κB signaling pathway, a key regulator of cell death responses. Statistics: data are expressed as mean ± standard deviation. Square brackets indicate statistically significant differences between experimental groups (*p* < 0.05). Parametric data: Caspase-3, cPARP, BCL2, pMLKL, NF-κB, LXRβ, TNFR1 (PC3), cGSDMD (PC3). Non-parametric data: TNFR1 (22Rv1), cGSDMD (22Rv1). Scale bars = 20 μm.

In 22Rv1 cells, activated caspase-3 and cPARP assays revealed induction of apoptosis (Figure 7A,B). All treatments increased active caspase-3 levels in 22Rv1 cells; however, the combinations with DHA did not change the effect of isolated drugs (Figure 7A). ENZ and iP monotherapies increased cPARP by 151% and ~91%, respectively, and DHA potentiated cPARP expression induced by iP, with a ~2-fold increase (Figure 7B). PC3 cells displayed a substantially attenuated apoptotic response compared to 22Rv1, with a distinct profile between the two markers analyzed (Figure 7C,D). All treatments induced a slight increase in activated caspase-3 in PC3 cells compared to control, and DHA potentiated the activation of caspase-3 when combined with iP (Figure 7C). In contrast, cPARP was not altered by DHA or iP monotherapies, but the DHA+iP combination reduced cPARP by ~59% compared to iP (Figure 7D), revealing divergent apoptotic mechanisms between 22Rv1 and PC3 cells.

Immunofluorescence analysis of the anti-apoptotic protein BCL2 demonstrated that in 22Rv1 cells, iP reduced BCL2 by ~21%, but the DHA+iP combination increased BCL2 by ~45% compared to iP. The DHA+ENZ combination also increased BCL2 by ~23% compared to control (Figure 7E,G). Conversely, in PC3 cells, all treatments reduced BCL2 expression compared to control. However, DHA+iP attenuated the maximal effect of iP monotherapy (Figure 7F,H).

### 2.10. DHA Triggers Pyroptosis and Potentiates iP-Induced Necroptosis in PC3 Cells

Recognizing that therapeutic resistance in CRPC often involves evasion of apoptosis, we next investigated whether DHA and its combinations with ENZ and iP engage alternative, non-apoptotic cell death pathways such as necroptosis (pMLKL, phosphorylated mixed-lineage kinase domain-like protein) and pyroptosis (cGSDMD, cleaved Gasdermin D).

In 22Rv1 cells, while ENZ induced a pronounced increase in pMLKL expression (141%), suggesting necroptosis activation, the DHA+ENZ combination resulted in reduced pMLKL expression (2.2-fold lower compared to ENZ), indicating that DHA antagonizes necroptosis in this cell line and may divert cell death toward alternative apoptotic pathways. iP alone or combined with DHA had no effect on necroptosis in 22Rv1 cells (Figure 7I). In PC3 cells, all treatments markedly increased pMLKL expression relative to control, with an apparent additive effect of DHA on iP-induced necroptosis (Figure 7J).

Regarding pyroptosis, in 22Rv1 cells, all treatments led to reduced expression of cGSDMD relative to control. In contrast, in PC3 cells, cGSDMD expression increased in the DHA (~38%) and DHA+iP (~33%) treatment groups compared to control, suggesting that DHA induces activation of pyroptosis in this cell line (Figure 7I,J).

### 2.11. DHA Increases TNFR1 Expression in Both 22Rv1 and PC3 Cell Lines

Because TNFR1 (Tumor Necrosis Factor Receptor Superfamily Member 1A) is a key upstream regulator of both apoptotic and necroptotic pathways, and LXRβ (Liver X Receptor beta) links pyroptosis, lipid metabolism, and survival signaling, we next examined how these receptors are modulated by DHA, ENZ, iP, and their combinations.

In 22Rv1 cells, TNF-R1 expression increased following DHA-containing treatments relative to the control. Furthermore, DHA potentiated TNFR1 upregulation compared with ENZ alone (Figure 7I). A similar pattern was observed in PC3 cells, in which DHA-containing treatments also increased TNFR1 expression (Figure 7J). LXRβ expression was reduced in the 22Rv1 cell line after treatment with the drugs alone or in combination with DHA but remained unchanged with DHA monotherapy. The DHA+ENZ combination caused a pronounced 3.4-fold reduction compared to ENZ (Figure 7I). In PC3 cells, LXRβ expression was not altered by the isolated treatments; however, the combination of DHA+iP resulted in a 71% decrease in expression compared with the control (Figure 7J).

### 2.12. iP Does Not Affect NF-κB in 22Rv1 Cells but Cooperates with DHA to Reduce NF-κB in PC3 Cells

Finally, because NF-κB (Nuclear Factor Kappa-Light-Chain-Enhancer of Activated B Cells) is a central mediator of pro-survival transcriptional programs in CRPC, we investigated whether NF-κB protein levels were modulated by DHA in a cell line-dependent manner. Integrating NF-κB expression with the observed changes in TNFR1 and cell death modalities was essential to understand how DHA reshapes stress and survival signaling under PARP and AR inhibition.

In 22Rv1 cells, NF-κB protein levels were reduced by 28%, 73% and 55% in the DHA, ENZ and DHA+ENZ groups, respectively, compared with the control, and the decrease induced by ENZ alone was partially attenuated in the combination. Treatments with iP did not alter NF-κB in this cell line (Figure 7I). In PC3 cells, only the DHA+iP combination reduced NF-κB (by 24%) relative to the control, and NF-κB protein levels in this group were approximately 1.4-fold lower than in cells treated with DHA or iP alone (Figure 7J).

## 3. Discussion

This study investigated the antitumor effects of the ω-3 PUFA docosahexaenoic acid (DHA) alone and in combination with ENZ or iP on 22Rv1 cells, and alone or in combination with iP on PC3 cells. Multiple mechanisms of DHA action previously described support the use of this ω-3 as a promising adjuvant antitumor agent in PCa, such as cytotoxic effects due to cell cycle arrest and apoptosis, increased oxidative stress, impaired mitochondrial function, and modulation of de novo lipid synthesis [19,20,21,24,25,26]. The novelty of this study is that DHA+iP demonstrated additive cytotoxicity, exhibiting an enhanced effect compared to monotherapy in PC3 cells related to suppression of the AKT proliferative pathway, reduced expression of pmTOR, ERα, PPARγ, LXRβ, and NF-κB, and increased necroptosis. This omega-3 also potentiated the cytotoxic effects of both ENZ and iP on 22Rv1 cells, impairing cell viability and colony growth while stimulating apoptosis and autophagy by modulating PPARγ and LXRβ. These insights position DHA as a versatile metabolic and signaling modulator that reconfigures the survival landscape of CRPC.

Our data demonstrate that DHA modulates the response to ENZ in 22Rv1 cells and the response to iP in both 22Rv1 and PC3 cells through treatment- and cell model-dependent mechanisms, particularly regarding cell death profiles and migration. PC3 cells, which are characterized by the loss of PTEN and AR, showed a pronounced response to PARP inhibitor monotherapy, since the compensatory DNA repair network and homologous recombination repair (HRR) in these cells are constitutively compromised, resulting in decreased cell proliferation and increased apoptosis [48,49]. On the other hand, the typical sensitivity of HRR deficient cells is not present in 22Rv1 cells because AR signaling maintains compensatory DNA repair. However, they remain responsive to PARP inhibition, likely due to their dependence on alternative repair pathways and the modulation of AR signaling [49,50].

As regards proliferative and cell survival pathways, all treatments resulted in reductions in PI3K and pAKT levels in both cell lines. Notably, DHA potentiated the action of iP by AKT pathway inactivation and mTOR downregulation specifically in PC3, but not in 22Rv1. The distinct sensitivity of PC3 can be attributed to its lack of PTEN expression, which leads to the hyperactivation of the PI3K-AKT-mTOR axis (as PTEN is a negative regulator of this pathway), making PC3 cells more dependent on this signaling for survival than 22Rv1 (PTEN^+^) [51]. It is well established that PI3K-AKT pathway hyperactivation reconfigures AR signaling requirements, maintaining AR-associated transcriptional programs even under low androgen deprivation. This adaptation occurs via AR cofactor modulation, activation of convergent signaling pathways on AR target genes, and cooperation with alternative mechanisms, ultimately contributing to castration resistance [47]. The downregulation of the AKT pathway corroborates the therapeutic importance of DHA in suppressing this hyperactivated axis in CRPC [47]. Mechanistically, DHA may alter membrane phospholipid composition and lipid raft organization, impairing the localization and activation of receptors and upstream kinases in the PI3K/AKT pathway, thereby reducing AKT phosphorylation, while effective PARP inhibition likely diminishes the reliance on AKT as a pro-survival pathway [52]. This combined effect may be an interesting therapeutic strategy, since AKT activation has been associated with resistance to PARP inhibitors [53].

Conversely, elevation of pERK1/2 in groups treated with iP may indicate compensatory ERK activation, representing an adaptive feedback mechanism well characterized in the oncology literature [54,55]. When PI3K/AKT signaling is inhibited, CRPC cells may activate compensatory crosstalk via MAPK/ERK as a survival evasion strategy [55]. This phenomenon suggests that isolated PI3K/AKT inhibition, even when robust, can be circumvented through activation of parallel survival pathways, thereby enabling therapeutic evasion. The increase in pERK1/2 activation with DHA+iP in PC3 cells suggests that AR-negative cells may be especially vulnerable to this escape mechanism [56]. Importantly, despite ERK activation, the combination of DHA and iP resulted in enhanced cytotoxicity, suggesting that other mechanisms override compensatory survival signaling, such as AKT as already discussed.

The DHA concentration used here caused robust clonogenic suppression in both cell lines, exceeding that induced by the individual drugs, and this effect was maintained in the combined treatments. Combined treatments with DHA not only reduced clonogenic survival but also diminished the intensity of the remaining colonies, reflecting impaired proliferative capacity in the surviving cells [57]. The marked inhibitory action of DHA on colony growth suggests lasting effects involving mechanisms that may not depend exclusively on the modulation of proliferative pathways, but may also include direct mitochondrial damage, ATP depletion, ROS overproduction and oxidative damage, reduced fatty acid synthase (FASN) expression and inhibition of de novo lipogenesis (DNL), induction of endoplasmic reticulum stress (ER stress), and mobilization of cell death pathways [17,19].

The additive effect of DHA on the cytotoxic response of PC3 cells to iP involved the downregulation of several nuclear receptors, including ERα, as well as lipid sensors such as PPARγ and LXRβ. ERα has a critical oncogenic role in CRPC, being highly expressed in these aggressive cells if compared to normal prostate and androgen-sensitive prostate cancer [58]. Estrogen-mediated ERα signaling promotes multiple androgen-independent malignant properties in CRPC, including proliferation, invasion, EMT, and formation of bone and lung metastases [59,60]. Thus, ERα signaling represents an important escape mechanism in CRPC, functioning as an alternative mediator of cell survival when androgen signaling is blocked. In this scenario, by negatively regulating ERα signaling the DHA+iP combination represents a promising strategy to block compensatory pathways in anti-androgen-resistant patients. In addition, the additive action of DHA on iP effect may also be due to DHA-derived metabolites presenting anti-inflammatory properties that may suppress estrogen signaling through altered NF-κB-ER crosstalk [25,61,62], as also demonstrated in this study.

PPARγ is considered a relevant regulator of lipid metabolism in PCa; however, its role in tumor development and progression appears to be highly context-dependent. Increased PPARγ expression or activity has been associated with lipogenic reprogramming, AR signaling, mitochondrial biogenesis, EMT, cell-cycle control, tumor growth, and metastatic progression; in addition, its expression is often increased in advanced, metastatic cases [63,64]. High PPARγ expression or elevated expression of PPARγ target genes is associated with more aggressive disease in several datasets [64,65]. Conversely, PPARγ activation has also been linked to cellular differentiation, growth inhibition, and tumor-suppressive responses [63,66]. These apparently contrasting functions may depend on the cellular background, disease stage, ligand availability, receptor isoform, and intracellular localization. In particular, PPARγ1 and PPARγ2 may regulate distinct metabolic and differentiation programs in prostate cells, with experimental evidence suggesting a more tumor-promoting role for PPARγ1 and a tumor-suppressive role for PPARγ2 [67]. Previous studies have reported heterogeneous PPARγ localization in prostate cancer tissues and cells, including predominantly nuclear or cytoplasmic expression patterns [68,69].

In this context, DHA emerges as a potential agent targeting multiple PPARγ-driven hallmarks. In the present study, iP reduced total PPARγ content in both cell lines, and DHA further enhanced this reduction under combined treatment. ENZ also reduced PPARγ content in 22Rv1 cells, in which this drug was evaluated. This amplifying effect of DHA on PPARγ downregulation under iP has important implications, as recent studies suggest that DHA may sensitize cells to death by suppressing PPARγ, thereby disrupting lipid-dependent metabolic survival programs and enhancing iP-induced replication stress [25]. Thus, these findings suggest that DHA may act as an adjuvant capable of modulating PPARγ-associated metabolic responses during iP treatment in CRPC. However, given the context-dependent functions of PPARγ, the biological significance of its reduced total content cannot be interpreted as exclusively tumor suppressive or directly linked to treatment sensitization. Further studies are needed to determine the contributions of PPARγ1 and PPARγ2, their intracellular localization and transcriptional activity.

LXRs also regulate lipid metabolism by controlling cholesterol homeostasis. Since LXRβ normally reduces intracellular cholesterol and inhibits survival pathways like AKT, its frequent downregulation in PCa promotes tumor survival [50]. Although reduced LXRβ expression observed with DHA+iP could be interpreted as unfavorable, evidence suggests that LXR activation can limit progression in advanced PCa via inhibition of EMT and metastasis through vimentin downregulation [70], which is consistent with our findings. In conclusion, the reduction in ER*α*, PPARγ and LXRβ by DHA under iP exposure could explain not only the negative modulation of AKT proliferative pathway and cell proliferation, but also other PCa hallmarks such as lipid metabolism and cell migration.

The 22Rv1 cell line expresses both AR-FL and the constitutively active AR-V7, a molecular hallmark of resistance to enzalutamide and clinical progression of CRPC [71]. Enzalutamide treatment markedly reduced AR-FL as expected [36] and was accompanied by a decrease in AR-V7 expression. Smaller reductions were observed after DHA, iP, and DHA+iP. ENZ and iP may contribute to the reduction in AR-V7 levels indirectly by modulating transcriptional programs and splicing regulation. In particular, therapy-induced cellular stress and DNA damage may interfere with the activity of kinase-regulated splicing factors, such as AKT, MEK/ERK, and YB-1, which are known to influence AR-V7 generation [72,73]. DHA-mediated AR-FL reduction is primarily due to the promotion of proteasomal degradation of the receptor and interference with its transactivation activity [74,75]. As mentioned, ENZ practically abolished both isoforms of AR in 22Rv1 cells, and the additive effect of DHA was not associated with further antiandrogenic effects, AKT pathway modulation, or additional impact on other nuclear receptors, except for LXRβ.

The additive effect of DHA on the ENZ response in 22Rv1 was not associated with an increase in the cell death programs investigated here, since apoptosis was unchanged, and LC3B accumulation and necroptosis were reduced in the DHA+ENZ group compared to ENZ alone. Thus, it is possible that this additive cytotoxic effect of DHA on the ENZ response is due to other mechanisms not analyzed here, such as induction of cell cycle arrest or impairment of cellular energy homeostasis, given that DHA impairs mitochondrial function, reduces ATP production, and promotes ROS accumulation and lipid peroxidation, effects previously reported for DHA [19,24,25,26].

GR plays a crucial role in the development of treatment resistance in CRPC. To bypass AR blockade, cancer cells can hyperactivate the GR, which compensates for the loss of AR signaling [76]. Thus, GR represents a potential therapeutic target for developing novel treatment strategies. All treatments reduced GR expression in both cell lines, without an additional effect when combined with DHA. The relative reduction in GR by ENZ in 22Rv1 cells reflects its ability to inhibit the compensatory feedback that normally occurs following AR blockade. Previous studies have already demonstrated the potential of DHA to reduce GR expression in prostate cells and other cell types [21,77]. The literature suggests that dual inhibition of AR+GR is more efficient than isolated AR blockade [78]. The maintenance of low GR levels by DHA combination treatments is a positive finding, because it may prevent a well-characterized mechanism of resistance to enzalutamide [79,80].

Here we confirmed that apoptosis is a common event triggered by drug treatments and DHA [27,36,39]. An additive effect of DHA on the apoptotic response induced by iP was observed in both cell lines. A central finding of this study is the DHA-induced upregulation of TNFR1 in both cell lines. TNFR1 is known to be a receptor capable of signaling toward cell survival, via NF-κB, which is inhibited by DHA [81], as well as toward apoptosis or necroptosis [82]. Our data suggest that DHA sensitizes both cell lines to cell death through increased TNFR1 expression, but the downstream molecular fate diverges: in 22Rv1 cells (partially functional p53, AR-positive), TNFR1 upregulation correlates with caspase-3 activation and cPARP, indicating that signaling was preferentially channeled toward canonical apoptosis, whereas in PC3 cells (p53-null, AR-negative), TNFR1 elevation combined with PARP inhibition (iP) resulted in increased activation of the necroptotic pathway (pMLKL), suggesting that the apoptotic pathway may have been overtaken by the faster kinetics of necroptosis. These divergent modes of regulated cell death suggest that AR status, together with the genetic background (for example, *TP53* status) and the lipid context, may contribute to the differential engagement of regulated cell death pathways in CRPC.

Necroptosis is a form of programmed cell death mediated by proteins such as RIPK1/3 (Receptor-Interacting Serine/Threonine-Protein Kinase 1/3) and MLKL, and represents a crucial alternative pathway when apoptosis is compromised [83]. As mentioned above, it occurs via activation of specific receptors such as TNFR (Tumor Necrosis Factor Receptor) or TLR (Toll-Like Receptors) under conditions of caspase inhibition [84]. It can be induced by TNFR1 activation in response to DNA damage [84]. It triggers necrosome formation, MLKL phosphorylation and activation, followed by its translocation to the plasma membrane, where it induces cell rupture [85]. In 22Rv1 cells, while ENZ alone induced necroptosis (increased pMLKL), DHA addition blocked this pathway (reduced pMLKL) and potentiated apoptosis (caspase-3/cPARP). We propose that this DHA-mediated blockade of necroptosis occurs through molecular crosstalk between caspase-8 and RIPK. The literature demonstrates that robust caspase-8 activation, necessary for the apoptosis observed in 22Rv1, prevents necrosome formation and necroptosis, and caspase-8-mediated cleavage of RIPK3 also restricts pyroptosis [86]. Thus, by sensitizing cells toward apoptosis, DHA indirectly suppresses the necroptotic pathway initiated by ENZ. However, the paradoxical BCL2 upregulation in the DHA+ENZ combination may reveal an adaptive resistance mechanism, wherein under severe stress, 22Rv1 cells may activate survival feedback loops in an attempt to evade cell death [87]. In PC3 cells, the DHA+iP combination induced an increase in pMLKL, indicating that necroptosis is the primary executor of cell death. The discussion in this cell line centers on the observation that caspase-3 was increased, yet cPARP was reduced in the DHA+iP combination relative to iP. While it deviates from the classical caspase-3/PARP cleavage model, it can be explained by necroptosis. Here, rapid cell death breaches membrane integrity before detectable cPARP accumulates or organelle rupture releases lysosomal proteases that degrade PARP non-specifically [88]. Moreover, given that cleaved caspase-3 is an early apoptotic marker, while PARP cleavage occurs at later stages of apoptosis, this temporal distinction may account for the observed discrepancy.

Pyroptosis is a programmed cell death characterized by the activation of inflammatory caspases (caspase-1, -4, -5, -11), which assemble the inflammasome. These caspases cleave Gasdermin D (cGSDMD), which in turn oligomerizes to form large pores in the plasma membrane, ultimately leading to cell death [83,85]. The most extensively studied inflammasome sensor is NLRP3 (NOD-like receptor family pyrin domain-containing 3), which can be activated by alterations in ionic flux, mitochondrial dysfunction, and other cellular stress signals [84]. DHA-induced mitochondrial dysfunction, characterized by hyperpolarization, ATP depletion, cristae damage, and increased reactive oxygen species (ROS) production, is a recognized trigger for NLRP3 activation and, in this context, may converge on caspase-1 activation and pyroptotic cell death [89]. Our data revealed cell line–specific differences in the modulation of pyroptosis. In 22Rv1 cells, all treatments reduced cGSDMD levels, suggesting suppression of pyroptosis. In contrast, in PC3 cells, DHA and DHA+iP increased cGSDMD levels, consistent with activation of this immunogenic form of cell death. These findings suggest that the molecular context of each cell line acts as a determinant of which regulated cell death pathway will be triggered [90].

The LC3B immunofluorescence signal was markedly increased across all treatments in 22Rv1 cells. In contrast, in PC3 cells, DHA and iP individually increased LC3B fluorescence, whereas the combination of DHA+iP reduced LC3B signal. Because LC3B was assessed under steady-state conditions without lysosomal blockade, these findings should not be interpreted as direct evidence of increased or decreased autophagic flux. Instead, they indicate treatment-associated changes in LC3B accumulation, which may result from alterations in autophagosome formation, maturation, or lysosomal degradation [91]. In the oncological context, autophagy may exert context-dependent functions, contributing to tumor suppression in early stages while supporting tumor cell survival and therapeutic resistance in established tumors. This functional ambiguity, balancing pro-survival and cell death mechanisms, makes its clinical targeting particularly complex, which explains why both autophagy inducers and inhibitors are being explored as therapeutic strategies [92]. In 22Rv1 cells, DHA attenuated enzalutamide-induced LC3B signal, which may reflect a shift in cellular response toward apoptotic pathways [93]. Although autophagy can act as a cytoprotective mechanism, its therapeutic induction can culminate in cell death when integrated with AR inhibitors or with modulation of regulators such as SKP2 [94]. This switching between autophagy and apoptosis is mediated by proteins such as Beclin-1, BCL2 and ATG5 (Autophagy Related 5), which function as crosstalk points between the two processes [95,96]. Additionally, PARPi can induce autophagy as a response to genomic stress in tumor cells, creating opportunities for therapeutic combinations with agents that sensitize cells to cell death [97].

Cell migration is a central component of tumor progression, closely linked to EMT, which promotes loss of cell adhesion and acquisition of invasive properties [98]. While the combination of DHA with iP demonstrated anti-migratory activity in PC3 cells, in 22Rv1 cells, ENZ, iP, and DHA monotherapies reduced wound closure, whereas DHA+ENZ and DHA+iP did not differ from the control. Therefore, the combined treatments did not retain the migration-inhibitory effect observed with the respective monotherapies. Recent in vitro studies demonstrate that DHA inhibits EMT through multiple mechanisms. This ω3 suppresses EMT markers, reduces mesenchymal gene expression, disrupts the TGF-β1/Smad signaling axis, and antagonizes GREM1-induced cell migration, thereby blocking both migration and invasion of cancer cells [99,100]. However, acute treatment with ENZ increases SNAIL expression [101,102]. Unlike DHA, which exhibits direct suppressive effects on EMT markers, the underlying mechanisms by which ENZ modulates EMT in CRPC remain incompletely characterized. There are limited data on the effects of PARPi on EMT, and no studies have specifically evaluated the PARP inhibitor AZD2461. A recent study demonstrated that clinically relevant PARPi, such as olaparib, rapidly reverse EMT markers, with restoration of E-cadherin expression and suppression of vimentin and fibronectin, while simultaneously reducing RAD51 (radiation sensitive 51 homolog) expression and promoting DNA damage, thus restoring sensitivity of tumor cells to genotoxic agents [103].

In 22Rv1 cells, DHA+ENZ increased E-cadherin expression, suggesting either a stabilizing effect on the epithelial phenotype or, alternatively, enhanced cell–cell adhesion that paradoxically reduces migration [98]. Vimentin expression was reduced under DHA+ENZ and DHA+iP treatment, which could be interpreted as a protective effect that reduces cellular plasticity and migratory capacity [98]. Although these changes are commonly associated with a more epithelial and less mesenchymal phenotype, they did not correspond to reduced wound closure in the combined treatment groups. This discordance indicates that modulation of individual EMT-related markers was insufficient to predict the functional response in this model. Moreover, the concurrent changes in epithelial- and mesenchymal-associated markers at the cell-population level may be consistent with heterogeneous or incomplete EMT-related alterations [104,105]. However, this interpretation remains speculative, and the present data do not allow definition of a specific transitional EMT state or identification of compensatory migration pathways. TGFβ-RII levels remained similar to the control in the combined treatment groups, despite the loss of the migration-inhibitory effect observed with the respective monotherapies. Therefore, TGFβ-RII expression alone was also insufficient to explain the wound-closure response. This observation highlights the clinically relevant fact that transcriptional EMT inhibitors may not translate into functional anti-migratory efficacy. In PC3 cells, a more mesenchymal-like model [70], the reductions in N-cadherin and vimentin under iP and DHA+iP treatment were more consistent with the delayed wound closure observed in these groups. Nevertheless, the heterogeneous responses of E-cadherin, SNAIL, and TGFβ-RII indicate that the treatments did not produce a uniform reversal of the EMT-associated phenotype. Collectively, these findings demonstrate treatment-associated modulation of EMT-related markers but do not establish complete EMT inhibition or identify the molecular mechanisms controlling migration in either cellular model.

## 4. Materials and Methods

### 4.1. Cell Culture and Maintenance

The following castration-resistant prostate cancer (CRPC) cell lines were used: 22Rv1 cells (#CRL-2505, American Type Culture Collection, Manassas, VA, USA) and PC3 cells (#CRL-1435, American Type Culture Collection, Manassas, VA, USA). Cells were cultured in RPMI 1640 medium (#R6504, Sigma-Aldrich, St. Louis, MO, USA) supplemented with 10% fetal bovine serum (FBS; #S0011, Vitrocell, Campinas, São Paulo, Brazil) and 1% penicillin, streptomycin, and amphotericin B (#15240062, Life Technologies, Paisley, UK) in a humidified incubator at 37 °C with 5% CO_2_. The culture medium was replaced every 2–3 days, and cells were subcultured upon reaching 70–85% confluence. For experiments, cells were seeded at the desired density and allowed to adhere for 24 h.

### 4.2. Cellular Treatment

DHA (#D2534—Sigma-Aldrich, St. Louis, MO, USA) was prepared at a concentration of 100 μM immediately before use in culture medium from a 20 mM stock solution in anhydrous ethanol (vehicle). The DHA concentration employed was selected based on previous studies from our research group [19,24,26,106]. Enzalutamide (ENZ; #S1250, Selleck Chemicals, Houston, TX, USA) working concentrations were prepared immediately before use from a 10 mM stock solution in dimethyl sulfoxide (DMSO; #D2650, Sigma-Aldrich, St. Louis, MO, USA; vehicle) [107], as well as the PARP inhibitor AZD2461 (iP; #SML1858, Sigma-Aldrich, St. Louis, MO, USA) [108,109]. For co-incubation experiments, DHA and the respective drug (ENZ or iP) at the desired concentration were added to the medium simultaneously. Vehicle concentrations in the medium did not alter cell viability.

### 4.3. MTS Assay for Estimation of Mitochondrial Metabolic Rate

Cell lines were seeded (5 × 10^4^ cells/well) in 96-well plates. Initially, 22Rv1 cells were treated with 100 μM DHA or with different concentrations of ENZ (10, 20, and 30 μM) and iP (5, 10, and 20 μM), administered either individually or in combination with DHA. The concentrations of ENZ and iP were below the estimated IC50 for these cell lines [107,108,109]. These sub-lethal concentrations of each drug as monotherapies were chosen to investigate whether their combination could trigger a powerful cytotoxic effect. PC3 cells were treated under the same DHA and iP conditions; however, ENZ was not evaluated because this cell line lacks AR expression and is therefore not an appropriate model for AR-targeted therapy. Accordingly, treatment effects were assessed within each cell line relative to its respective control, rather than through a direct comparison between the two cell lines. The effects of 24 and 48 h incubations on mitochondrial metabolic rate (MMR) [24] were evaluated using the 3-(4,5-dimethylthiazol-2-yl)-5-(3-carboxymethoxyphenyl)-2-(4-sulfophenyl)-2H-tetrazolium (MTS) assay, performed with the commercial colorimetric CellTiter 96^®^ AQueous One Solution Cell Proliferation Assay (#G3580—Promega Corporation, Madison, WI, USA), according to the manufacturer’s instructions. In metabolically active cells, MTS is bioreduced by NAD(P)H-dependent dehydrogenase enzymes into a soluble formazan product. The CellTiter 96^®^ AQueous One Solution Reagent was added directly to culture wells, followed by a 1 h incubation before absorbance was measured at 490 nm using an Epoch microplate reader (BioTek Instruments Inc., Winooski, VT, USA). The absorbance measured at 490 nm is directly proportional to the amount of soluble formazan produced and was used as an indicator of mitochondrial metabolic activity. Four independent experiments, each performed in triplicate, were conducted for statistical analysis (*n* = 4). Values were expressed as fold-change relative to control. The concentration and exposure time of ENZ and iP that most significantly altered MMR were selected for all downstream assays (ENZ: 30 μM; iP: 20 μM; 48 h).

### 4.4. Cell Viability and Proliferation Assays

#### 4.4.1. Trypan Blue Exclusion Assay

Following treatments, cells were collected by trypsinization and resuspended in culture medium. A 20 μL aliquot of the cell suspension was mixed with 20 μL of 0.4% Trypan Blue solution (#15250-061, Thermo Fisher Scientific, Waltham, MA, USA) in a 1:1 ratio. Cell counting was performed using a Neubauer chamber under an inverted optical microscope (Eclipse TS100, Nikon Corporation, Tokyo, Japan). Viable cells (unstained, with intact membrane) and dead cells (stained blue, with permeabilized membrane) were counted in four quadrants of the Neubauer chamber. Total cell number and percentage of cell viability were calculated as follows: cell density (cells/mL) = (number of cells counted/number of quadrants) × dilution factor × 10^4^; viability (%) = (viable cells/total cells) × 100 [110]. Results were expressed as mean ± standard deviation. Four independent experiments performed in duplicate were conducted for statistical analysis (*n* = 4).

#### 4.4.2. Automated DAPI-Stained Nuclei Counting

To analyze cell number following the treatments, automated nucleus counting was performed using 4′,6-diamidino-2-phenylindole (DAPI) staining. Cells were seeded (1.5 × 10^4^ cells/well) in 96-well plates. Following treatments, cells were fixed with 4% paraformaldehyde for 10 min at room temperature. Subsequently, wells were washed with phosphate-buffered saline (PBS) and cells were stained with DAPI (0.5 μg/mL; #D9542, Sigma-Aldrich) for 10 min at room temperature protected from light. Image acquisition was performed using the Operetta High-Content Imaging System (PerkinElmer, Shelton, CT, USA). Wells were fully imaged in the DAPI fluorescence channel (Ex 358 nm/Em 461 nm). Analysis of the total number of nuclei per well was based on automated counting using Columbus software (version 2.4.0.104236, PerkinElmer, Shelton, CT, USA) with a nuclear segmentation algorithm based on circularity and fluorescence intensity. Results are expressed as the percentage of nuclei relative to control, with reduced values indicating a lower cell number, which may reflect reduced proliferation and/or treatment-induced cell loss. Four independent experiments performed in triplicate were conducted for statistical analysis (*n* = 4).

### 4.5. Colony Formation Assay

Cells were seeded (1 × 10^3^ cells/well) in 6-well plates. Following cell adhesion, cells were subjected to treatments for 48 h. Subsequently, the medium containing treatments was removed, cells were washed with PBS, and fresh RPMI medium supplemented with 10% FBS without treatments was added. Cells were then incubated for 14 days to allow colony formation, with medium replacement every 3–4 days. At the end of the incubation period, cells were fixed with 50% methanol, stained with 0.5% Crystal Violet for 20 min, followed by washing with distilled water until complete removal of background dye [111]. Colony formation was quantified using the ColonyArea plugin in ImageJ software (version 1.54g; National Institutes of Health, Bethesda, MD, USA) [112]. Data are expressed as colony area, which indicates the percentage of well area covered by colonies, and colony intensity, a parameter that accounts for both the area covered by colonies and colony density (staining intensity, reflecting the number of cells per colony). Experiments were performed independently four times, in duplicate (*n* = 4).

### 4.6. Scratch Assay

Cell migration capacity under the treatments tested was evaluated using a cell scratch/wound healing assay. Cells (1 × 10^6^) were seeded in 6-well plates (*n* = 3 independent replicates per condition) and cultured until 90–100% confluence was achieved. Upon reaching confluence, the culture medium was removed, wells were washed with sterile PBS to remove suspended cells, and a linear scratch was made across the monolayer using a sterile P200 pipette tip along the well diameter. Each well received a single central scratch. Cell debris was removed by PBS washes, and cells were treated and photographed immediately (0 h). Images of the same field in each well were obtained at 12, 24, 36, and 48 h using a 10× objective. For each well, five fields were photographed at each time point. Subsequently, images were analyzed using ImageJ software (version 1.54g; National Institutes of Health, Bethesda, MD, USA) by measuring the scratch area over time. For each treatment, the percentage of wound closure at each time point was calculated according to the formula: % wound closure = [(area 0 h − area t)/area 0 h] × 100. Closure of the scratched area represents the migratory movement of cells from the wound edge toward the scratch center [113].

### 4.7. Immunofluorescence Assay for LC3B and BCL2

The immunofluorescence assay was used to evaluate the expression of microtubule-associated protein 1 light chain 3B (LC3B), a marker of autophagosomes/autophagy, and B-cell lymphoma 2 (BCL2), which is associated with apoptosis resistance and autophagy. Cells were seeded (3 × 10^5^ cells/well) on sterile glass coverslips in 6-well plates. Following treatments, culture medium was aspirated, and cells were washed with Tris-buffered saline with Tween 20 (TBS-T). Fixation was performed with 4% paraformaldehyde for 10 min at room temperature. Subsequently, cells were incubated with 100 mM glycine for 5 min and washed with TBS-T. Permeabilization was carried out with 0.2% Triton X-100 in TBS-T for 10 min on ice, followed by three washes with TBS-T. Blocking of nonspecific binding was performed with 3% bovine serum albumin (BSA) for 1 h at room temperature. Cells were then incubated with primary antibodies anti-BCL2 (1:100 in 1% BSA, #3498, rabbit monoclonal, Cell Signaling, Danvers, MA, USA) or anti-LC3B (1:100 in 1% BSA, #43566, rabbit monoclonal, Cell Signaling, Danvers, MA, USA) for 1 h at room temperature. Following three washes with TBS-T (5 min each), incubation was performed with anti-rabbit IgG-FITC (#F0382, 1:200 in 1% BSA, Sigma-Aldrich, St. Louis, MO, USA) for 1 h at room temperature protected from light. Finally, coverslips were washed with TBS-T and mounted with DAPI-containing mounting medium (Fluoroshield™ with DAPI, #F6057, Sigma-Aldrich, St. Louis, MO, USA).

Images were acquired using an AX10 fluorescence microscope (Zeiss, Oberkochen, Germany) coupled with AxioVision software (version 4.8.2.0; Zeiss, Oberkochen, Germany). For each experimental condition, 3 slides from different passages were analyzed and at least 15 microscopic fields per slide were evaluated, totaling 45 areas analyzed per group. Negative controls were performed by omitting the primary antibody. Results are expressed as relative fluorescence, calculated as the ratio between the mean fluorescence intensity of the cytoplasmic protein of interest and the mean nuclear fluorescence intensity in the analyzed fields, using ImageJ software (version 1.54g; National Institutes of Health, Bethesda, MD, USA).

### 4.8. Evaluation of Active Caspase-3

Caspase-3 proteolytic activity was determined using a fluorescence-based assay with the EnzChek^®^ Caspase-3 Assay Kit #1 with Z-DEVD-AMC substrate (#E13183—Thermo Fisher Scientific, Waltham, MA, USA), according to the manufacturer’s specifications. Cells, cultured in 6-well plates (5 × 10^5^ cells/well), were collected following treatments and lysed in RIPA buffer supplemented with 10% protease inhibitor cocktail (#P8340), phosphatase inhibitors (#P5726), and Triton X-100. All reagents were obtained from Sigma-Aldrich (St. Louis, MO, USA). Lysates were then incubated on ice for 2 h and centrifuged at 14,000 rpm for 20 min at 4 °C. Total protein in the supernatant was quantified using the Bradford method [114] and measured using an Epoch microplate reader (BioTek Instruments Inc., Winooski, VT, USA) at 595 nm. Subsequently, aliquots containing 30 μg of protein were pipetted into 96-well dark plates, and the Z-DEVD-AMC substrate, diluted in reaction buffer, was added to each sample. The plate was incubated for 30 min at room temperature. Following reaction completion, active caspase-3 levels were measured using a fluorescence microplate reader (Molecular Devices, Menlo Park, CA, USA) at Ex 342 nm/Em 441 nm. Fluorescence is a direct measure of the quantity of active caspase-3 based on an AMC standard curve. Controls with Ac-DEVD-CHO were used to ensure specificity, according to the manufacturer’s guidelines. The experiment was performed in duplicate using cell lysates from 4 independent experiments per group (*n* = 4).

### 4.9. Western Blotting

Following treatments, cells cultured in 6-well plates (5 × 10^5^ cells/well) were lysed in RIPA extraction buffer supplemented with 10% protease inhibitor cocktail (#P8340) and phosphatase inhibitor (#P5726) (Sigma-Aldrich, St. Louis, MO, USA), followed by mechanical disruption. Lysates were incubated on ice for 2 h and subsequently centrifuged at 14,000 rpm for 20 min at 4 °C to remove cell debris. Total protein quantification in samples was performed using the Bradford method [114] and measured using an Epoch microplate reader (BioTek Instruments Inc., Winooski, VT, USA) at 595 nm. Aliquots containing 20 μg of protein were separated by sodium dodecyl sulfate polyacrylamide gel electrophoresis (SDS-PAGE) and, following electrophoresis, transferred for 2 h to a nitrocellulose membrane (Amersham Life Science, Slough, BU, UK) using a wet transfer system. Blocking of nonspecific binding for phosphorylated proteins was performed with 5% BSA in TBS-T for 1 h at room temperature, whereas for other proteins 5% nonfat milk in TBS-T was used for 30–60 min at room temperature. Primary antibodies were incubated overnight at 4 °C (details—name, dilution, characteristics, and catalog number/manufacturer—in Table S1 in Supplementary Material). Subsequently, membranes were incubated for 1 h at 4 °C with secondary antibodies anti-rabbit-HRP (#sc-2357) or anti-IgGκ-HRP (#sc-516) (Santa Cruz Biotechnology, Dallas, TX, USA), prepared at a 10-fold dilution relative to the respective primary antibody dilution. Detection was performed by enhanced chemiluminescence (ECL) and bands were visualized using the G:BOX Chemi XRQ system (Syngene, Synoptics, Cambridge, UK). Relative densitometry was evaluated using ImageJ software (version 1.54g; National Institutes of Health, Bethesda, MD, USA) following normalization to GAPDH, β-actin, or the total form of the protein of interest. All assays were performed in four independent experimental replicates (*n* = 4).

### 4.10. Statistical Analyses

Quantitative data were subjected to statistical analyses using GraphPad Prism software (version 8.0.1; GraphPad Software, Boston, MA, USA). Normality was assessed using the Shapiro–Wilk test. Parametric data were subjected to one-way analysis of variance (ANOVA) followed by Tukey’s multiple comparison test. Non-parametric data were analyzed using the Kruskal–Wallis test with Dunn’s post hoc test. Statistical comparisons were made between groups: control, DHA, drug (ENZ or iP), and DHA + drug; in the 22Rv1 cell line, ENZ and iP were not compared with each other. Results are presented as mean ± standard deviation, and *p* < 0.05 was considered statistically significant.

## 5. Conclusions

The present study demonstrates that DHA exerts significant antitumoral effects as monotherapy in CRPC cell models and holds potential clinical applicability as an adjuvant strategy; however, its interactions with targeted therapies (enzalutamide and the PARP inhibitor AZD2461) are complex and highly dependent on molecular context. The initial hypothesis that DHA, in combination with enzalutamide and the PARP inhibitor (AZD2461), would potentiate cytotoxic effects through mechanisms distinct from those of the individual drugs was confirmed in a setting dependent on AR status. In PC3 cells (AR−), the DHA+iP combination exhibited additive cytotoxic effects associated with reductions in pAKT and pmTOR levels and the downregulation of ERα, as well as nuclear receptors involved in lipid metabolism such as PPARγ and LXRβ. DHA also reshaped the cell death profile triggered by iP, promoting a shift toward apoptosis together with robust necroptosis activation, consistent with a more immunogenic pattern of regulated cell death. In 22Rv1 cells (AR+), DHA in combination with ENZ or iP likewise enhanced cytotoxicity in a different way. The DHA+ENZ combination reduced LC3B accumulation, increased TNFR1 expression, and decreased LXRβ expression, whereas DHA+iP reduced PPARγ expression and enhanced apoptosis. Collectively, these observations place DHA as a promising adjuvant candidate in CRPC, particularly in iP-treated PC3 cells, which exhibited a consistent positive response across multiple cytotoxic, apoptotic and alternative regulated cell death parameters. In 22Rv1 cells, although DHA displays cytotoxic effects and enhances drug-induced apoptosis, issues related to cell migration and EMT heterogeneity require further evaluation to optimize therapeutic protocols.

In the AR-negative PC3 cell model, the DHA+iP combination is a rational strategy that concomitantly activates necroptosis and pyroptosis. This reinforced state of immunogenic cell death could enhance in vivo responses in immunocompetent tumors by promoting lymphocytic infiltration and boosting the efficacy of immune checkpoint inhibitors. Furthermore, this approach represents an opportunity to target two parallel vulnerability axes: replication stress driven by PARP inhibition and lipid-directed metabolic reprogramming in tumors that lack endocrine adaptive escape routes (AR/ER). Future studies directly comparing different treatment sequences, exposure durations, and dosing schedules are required to define the most effective combination strategies. Validation in additional CRPC models and in vivo studies will also be necessary before the potential therapeutic relevance of these combinations can be established.

## Data Availability

The original contributions presented in this study are included in the article/Supplementary Materials. Further inquiries can be directed to the corresponding author.

## Supplementary Materials

The following supporting information can be downloaded at: https://www.mdpi.com/article/10.3390/ijms27188037/s1.
