# Supplementary material for: Docosahexaenoic Acid Enhances the Cytotoxic Effects of Enzalutamide and the PARP Inhibitor in Castration-Resistant Prostate Cancer Cell Lines
Source: Int J Mol Sci. 2026 Sep 9;27(18):8037. doi: 10.3390/ijms27188037 (PMC13607705; doi:10.3390/ijms27188037)
Supplement: Supplementary file 1 [file ijms-27-08037-s001.zip › ijms-4401163-supplementary.pdf]

Supplementary material

Table S1 – Information on the primary antibodies used in the Western blotting assays.

Antibody	Source	Epitope specificity	Supplier	Code	Dilution
**AKT**	Rabbit	Polyclonal	Immuny	IM0051	1:500 in BSA 1%
**cGSDMD**	Rabbit	Monoclonal	Cell Signaling Technology	#10137	1:500 in BSA 1%
**cPARP**	Rabbit	Monoclonal	Cell Signaling Technology	#5625	1:700 in BSA 1%
**E-cadherin**	Rabbit	Monoclonal	Cell Signaling Technology	#3195S	1:500 in BSA 1%
**ERK1/2**	Rabbit	Polyclonal	Cell Signaling Technology	#9102	1:500 in BSA 1%
**ER-α**	Mouse	Monoclonal	Santa Cruz Biotechnology	sc-8005	1:200 in BSA 1%
**GAPDH**	Mouse	Monoclonal	Sigma-Aldrich	G8795	1:10.000 in BSA 1%
**GR**	Rabbit	Polyclonal	Santa Cruz Biotechnology	sc-1004	1:400 in BSA 1%
**LC3B**	Rabbit	Monoclonal	Cell Signaling Technology	#43566	1:200 in BSA 1%
**LXR-β**	Rabbit	Monoclonal	Cell Signaling Technology	#13519	1:500 in BSA 5%
**NF-κB**	Rabbit	Monoclonal	Cell Signaling Technology	#8242	1:500 in BSA 1%
**N-cadherin**	Rabbit	Monoclonal	Cell Signaling Technology	#13116S	1:500 in BSA 1%
**pAKT**	Rabbit	Polyclonal	Santa Cruz Biotechnology	sc-293125	1:400 in BSA 1%
**pERK1/2**	Rabbit	Polyclonal	Cell Signaling Technology	#4370S	1:400 in BSA 1%
**PI3K**	Rabbit	Polyclonal	Immuny	-	1:500 in BSA 1%
**pMLKL**	Rabbit	Monoclonal	Cell Signaling Technology	#37333	1:500 in BSA 1%
**pmTOR**	Rabbit	Monoclonal	Cell Signaling Technology	#5536	1:500 in BSA 1%
**PPARγ**	Rabbit	Polyclonal	Cell Signaling Technology	#2430S	1:500 in BSA 1%
**SNAIL**	Rabbit	Monoclonal	Cell Signaling Technology	#3879S	1:350 in BSA 1%
**TGFβ-RII**	Rabbit	Monoclonal	Cell Signaling Technology	#3713S	1:500 in BSA 1%
**TNF-R1**	Rabbit	Polyclonal	Santa Cruz Biotechnology	sc-7895	1:200 in BSA 1%
**Vimentin**	Mouse	Monoclonal	-	-	1:400 in BSA 1%
**B-actin**	Mouse	Monoclonal	Santa Cruz Biotechnology	sc-47778	1:500 in BSA 1%

Source: Prepared by the author.
